# Toothbrushing effects on the surface roughness and cement volume loss of bonded enamel-ceramic interface

**DOI:** 10.1590/0103-6440202305459

**Published:** 2023-12-22

**Authors:** Luísa de Almeida Vieira Marins, Vitaliano Gomes Araújo-Neto, Beatriz Ometto Sahadi, Carolina Bosso André, Marcelo Giannini

**Affiliations:** 1 Piracicaba Dental School, University of Campinas; Piracicaba, SP, Brazil; 2 Dental Materials Division, Department of Restorative Dentistry, Piracicaba Dental School, University of Campinas; Piracicaba, SP, Brazil; 3 Operative Dentistry Division, Department of Restorative Dentistry, Piracicaba Dental School, University of Campinas; Piracicaba, SP, Brazil; 4 Operative Dentistry Division, Department of Restorative Dentistry, School of Dentistry, Federal University of Minas Gerais; Belo Horizonte, MG, Brazil

**Keywords:** Resin cement, resin composite, dental enamel, dental ceramic, toothbrushing

## Abstract

This study evaluated the effect of toothbrushing on enamel-cementing material-ceramic bonded interfaces, using different cementing materials. Materials and Methods: Thirty enamel and thirty ceramic blocks were bonded with cementing materials to produce the samples that were bonded with three types of cementing materials: 1- RelyX Ultimate resin cement (REXU), 2- RelyX Unicem 2 self-adhesive resin cement (REU2) and 3- heated Z100 restorative composite (60°C). Bonded interfaces of the samples were toothbrushed and the surfaces of the 3 cementing materials were evaluated for roughness (RG, in µm), roughness profile (RP, in µm), and volume loss (VL, in µm3) (baseline and after 20,000 and 60,000 toothbrushing cycles). Data were evaluated by Generalized Linear Analysis (two factors: “material” and “toothbrushing cycle”) and Bonferroni test (α=0.05). Results: REXU and Z100 exhibited lower RG than that presented by REU2, except after 60,000 toothbrushing cycles when only Z100 differed from REU2. The increase in toothbrushing cycles increased the RG and RP for all materials. REU2 also showed higher RP than those showed by REXU and Z100 when it was analyzed regarding the enamel. The VL of Z100 was the lowest with 20,000 toothbrushing cycles, regarding the enamel and ceramic. For 60,000 cycles, REXU showed the lowest VL regarding the ceramic, and REU2 had the highest VL regarding the enamel and ceramic. Conclusion: In general, REXU and Z100 showed the best results regarding the evaluations performed and the REU2 exhibited the highest RG, RP, and VL.

## Resumo

Este estudo avaliou o efeito da escovação nas interfaces de união esmalte-material cimentante-cerâmica, utilizando diferentes materiais cimentantes. Materiais e Métodos: Trinta esmaltes e trinta blocos cerâmicos foram unidos com materiais de cimentação para produzir as amostras que foram unidas com três tipos de materiais de cimentação: 1- cimento resinoso RelyX Ultimate (REXU), 2- cimento resinoso autoadesivo RelyX Unicem 2 (REU2) e 3-compósito restaurador aquecido Z100 (60°C). As interfaces coladas das amostras foram escovadas e as superfícies dos 3 materiais cimentantes foram avaliadas quanto à rugosidade (RG, em µm), perfil de rugosidade (RP, em µm) e perda de volume (VL, em µm3) (baseline e após 20.000 e 60.000 ciclos de escovação). Os dados foram avaliados por Análise Linear Generalizada (dois fatores: “material” e “ciclo de escovação”) e teste de Bonferroni (α=0,05). Resultados: REXU e Z100 apresentaram menor RG do que REU2, exceto após 60.000 ciclos de escovação, quando Z100 diferiu apenas de REU2. O aumento dos ciclos de escovação aumentou o RG e RP para todos os materiais. O REU2 também apresentou PR maior que o REXU e Z100, quando analisado em relação ao esmalte. O VL de Z100 foi o menor com 20.000 ciclos de escovação em relação ao esmalte e cerâmica. Para 60.000 ciclos, o REXU apresentou o menor VL em relação à cerâmica e o REU2 o maior VL em relação ao esmalte e à cerâmica. Conclusão: De maneira geral, REXU e Z100 apresentaram os melhores resultados nas avaliações realizadas e o REU2 apresentou os maiores RG, RP e VL..

## Introduction

The procedure for bonding resin cement to dental ceramics plays a fundamental role in the clinical longevity of indirect restorations. Following the advent of all-ceramic materials, resin cements gained popularity for presenting different activation modes, good adhesion to the tooth structure, and aiming esthetics after cementation, Thus, resin cements are the main cementing material for prosthetic restorations in anterior and posterior teeth ^1^.

Supragingival margin preparations for indirect restorations have advantages, such as facilitating impression, cementation, and biofilm control at the tooth-restoration interface ^2^. However, the restoration-tooth interface at supragingival margins is directly affected by the toothbrushing, because the Bass toothbrushing technique preconizes brushing thoroughly around and under the gumline, where biofilm tends to accumulate ^3^ in order to control and prevent periodontal and caries diseases. Thus, the Bass toothbrushing technique might lead to degradation and removal of the resin cement layer at supragingival margin preparations for indirect restorations. Also, the shrinkage stress generated during the polymerization reaction can debond the resin cement to ceramic or tooth, damaging the adhesive interface by the gap formation ^4^.

The mechanical properties of the resin cement are inferior compared with ceramics and dental enamel, making it the most critical region in the cementing area. In cases of poor adaptation of the indirect restoration to the cavity margins, the resin cement can be thick, compromising the marginal sealing of tooth structure ^1^,^4^,^5^.

Dental resin cements are classified according to their activation mode and bonding mechanism. Regarding the activation mode, dual-cured resin cements have been widely used, because they have indications in several types of cementations. The bonding mechanism of self-adhesive resin cement differs from the conventional cementing system because they do not require the application of etchants, adhesives, or primers to the tooth structure for bonding the indirect restorations ^6^,^7^. The adhesive properties of self-adhesive resin cement depend on the addition of adhesion-promoter components, such as esters, to the traditional resinous matrix of resin cement. In addition, they contain glass powder and organic acids that can develop an acid-base reaction. These compositional changes may alter the chemical and mechanical properties of self-adhesive resin cement ^1^,^6^ ^), (^ ^7^ ^),(^ ^8^.

An alternative to improve the marginal integrity of indirect restorations is the use of pre-heated restorative resin composites as cementing materials. The heated composite presents low viscosity that allows the cementation procedure ^9^, ^(^ ^10^ ^), (^ ^11^ ^), (^ ^12^ ^), (^ ^13^ ^),(^ ^14^. The dentists have used these varieties of cementing materials, but different compositions of materials might lead to different clinical performance regarding the chemical and mechanical challenges in the oral cavity ^1^,^6^,^13^.

This study evaluated the effect of toothbrushing on three types of cementing materials: one conventional dual-cured cementing system, one self-adhesive dual-cured resin cement, and one heated, light-cured restorative resin composite. The research hypotheses were that: 1- simulated toothbrushing does not affect the roughness surface of cementing materials; 2- the roughness profile varies among cementing materials after toothbrushing, and 3- the toothbrushing cycles induce the volume loss of these materials.

## Materials and methods

### Specimen Preparation, Experimental Groups, and Toothbrushing

The materials used in the study and their respective compositions are shown in Box 1. The following materials were used: a feldspathic ceramic (Cerec Blocs, Dentsply Sirona, Bensheim, Germany), two etchants: 35% phosphoric acid (Gluma Etch 35, Kulzer GbmH, Hanau, Germany), and 5% hydrofluoric acid (Maquira Dental Group, Maringá, PR, Brazil), a silane coupling-agent (Ceramic Primer, 3M Oral Care, St. Paul, MN, USA), a bonding agent (Scotchbond Universal, 3M Oral Care), two resin cement: RelyX Ultimate (REXU, 3M Oral Care) and RelyX Unicem 2 (REU2, 3M Oral Care) and one restorative resin composite (Z100, 3M Oral Care).

**Box 1:**
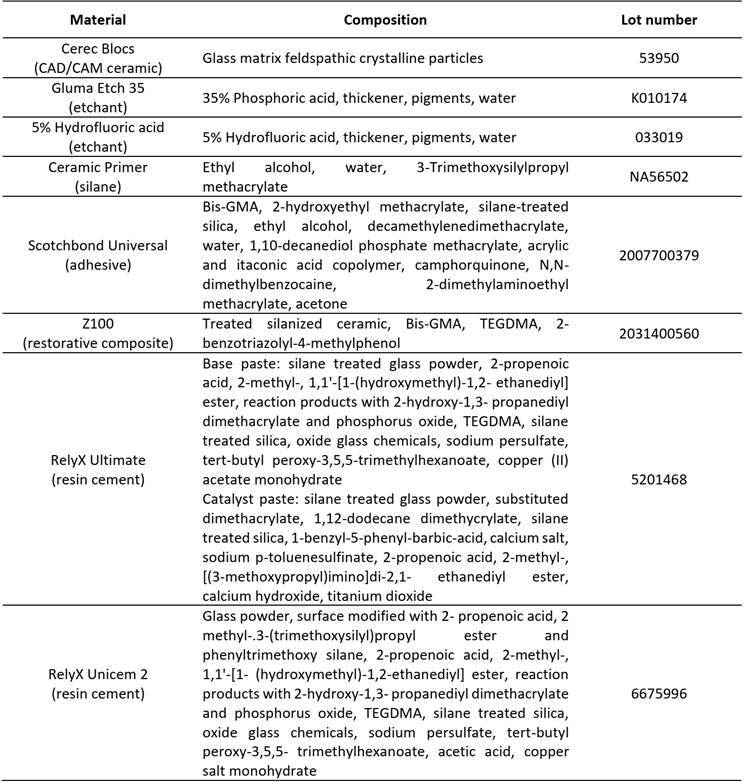
Compositions and lot numbers of the dental materials used in this study.

Monomer abbreviations: Bis-GMA: Bisphenol A diglycidyl ether dimethacrylate; TEGDMA: Triethylene glycol dimethacrylate.

Thirty incisor bovine teeth were used for this study. The roots of the teeth were removed and 30 enamel blocks (3 mm thick x 5 mm wide x 5 mm long) were obtained by sectioning the teeth with a diamond disc (Buehler Ltd., Lake Bluff, IL, USA). The buccal enamel was flattened with 600-grit sandpaper (Norton Abrasivos, Guarulhos, SP, Brazil) under water irrigation. Thirty ceramic blocks with the same dimensions as the enamel blocks were obtained from the CAD/CAM Cerec Blocs. The three types of cementing materials formed the three experimental groups for this study: REXU, REU2, and Z100.

Ceramic blocks were etched with 5% hydrofluoric acid for 60 seconds, washed with water for 30 seconds, and subjected to an ultrasonic bath in an ultrasonic cleaner (USC 1400; Unique, Indaiatuba, SP, Brazil), containing distilled water for 5 minutes, followed by air-drying. The etched ceramic surfaces were silanated (Ceramic Primer, 3M Oral Care) only for REU2 and Z100 followed by adhesive application (Scotchbond Universal) that was kept uncured. For REXU, the Scotchbond Universal adhesive was directly applied to etched ceramic without silanization and also kept uncured, according to the manufacturer's instructions.

For the cementation with Z100 and REXU, the dental enamel surfaces were etched with 35% phosphoric acid for 20 seconds, washed with water for 30 seconds, and air-dried. Scotchbond Universal adhesive was applied to etched, dry enamel, keeping the adhesive uncured as those applied to enamel block. The acid-etching mode was chosen for this universal adhesive to ensure higher bond strength ^15^. For REU2, no bonding agent was used and the enamel was not etched, keeping it untreated as recommended by its manufacturer.

When using Z100, it was heated until 60°C before cementation ^9^, using a dental composite heating device (HotSet, Technolife, Joinville, SC, Brazil), while REXU and REU2 were used at room temperature (23°C). The cementing materials were manipulated and applied to the silanated and bonded ceramic surface, which was then placed on the enamel surface with a 500 g load. The excess cementing materials were removed with microbrush disposable applicators, and they were light-cured from all surfaces for 20 seconds (1,474 mW/cm^2^, Valo Cordless, Ultradent Products Inc., South Jordan, UT, USA).

Bonded samples were stored in deionized water for 24 hours at room temperature, embedded in epoxy resin (Buehler Ltd.) and the ceramic-cementing material-enamel interfaces were polished with sandpapers (800, 1000, 1200, and 2000-grits, Norton Abrasivos) under water irrigation and diamond paste (1 μm, Buehler Ltd.).

Samples were submitted to 20,000 and 60,000 cycles (150 cycles/min) of toothbrushing (MEV-4X 3D, Odeme Dental Research, Luzerna, SC, Brazil). A 200 g load was delivered by soft toothbrushes (Oral-B Indicator 35, Procter & Gamble, Seropédica, RJ, Brazil) and samples were covered in a slurry of toothpaste (Oral-B Pro-Health, Procter & Gamble) solution (16 g of dentifrice with 100 mL of deionized water). After each simulated toothbrushing cycle, the samples were thoroughly washed and air-dried before being analyzed.

### Confocal Microscopy Analysis

The surface roughness (Sa, in μm), roughness profile (Rv, in μm), and volume loss (μm^3^) were measured using confocal microscopy (LEXT 3D Measuring Laser Microscope OLS4000, Olympus Corp., Tokyo, Japan) and the OLS4000 software (Olympus Corp). The Sa parameter describes the arithmetic height deviation from a mean plane three-dimensionally and corresponds to the two-dimensional parameter Ra, which measures surface roughness by detecting the maximum peak-to-valley heights of a specific surface profile. The surface roughness of cementing materials was measured at the baseline (unbrushed) and after 20,000 and 60,000 toothbrushing cycles.

An image containing 0.5 mm of the ceramic or enamel sides of each sample was obtained to calculate the roughness profile and the volume loss. The roughness profile was determined from the largest valley depth deviation from the mean line within a given length (10 readings at each image). To calculate the volume loss, a reference plan from the top of the ceramic or enamel area was defined and the software calculated the volume loss of cementing material located below this reference. For the roughness profile, its was considered the measurements obtained at baseline (unbrushed) and after 20,000 and 60,000 toothbrushing cycles. For the volume loss analysis, only the toothbrushing cycles were considered, and the height of the enamel and ceramic as a reference for calculating the volume loss of the cementing materials. Surface roughness, roughness profile, and volume loss data were analyzed by Generalized Linear Analysis (two factors: “cementing material” and “toothbrushing cycles") and the Bonferroni test (α = 0.05).

Representative 3D images including the cementing materials, ceramic, and enamel were obtained to visualize the interfacial structures of samples 60.000 after toothbrushing cycles. A 5x objective lens (1x zoom) was used to obtain the images (1024 x 1024 pixels, XYZ fast scan) with a 405 nm laser (Gaussian filter). The thickness of resin cement layers was calculated using the scales of 3D images.

## Results

### Surface Roughness

Table 1 presents the surface roughness results for the cementing materials at baseline, 20.000, and 60.000 toothbrushing cycles. REXU and Z100 showed lower surface roughness than that obtained for REU2 at baseline and 20.000 toothbrushing cycles. However, only Z100 differed from REU2 at 60.000 cycles. The increase in the number of toothbrushing cycles increased the surface roughness for the three cementing materials.

**Table 1:** Surface roughness means (Sa, in µm) for the cementing materials (95% CI).

Material	Baseline	20,000 cycles	60,000 cycles
REXU	0.12 (0.08 - 0.17) bC	0.26 (0.17 - 0.34) bB	0.67 (0.45 - 0.89) abA
REU2	0.32 (0.21 - 0.42) aB	0.49 (0.33 - 0.65) aB	1.00 (0.67 - 1.33) aA
Z100	0.11 (0.07 - 0.14) bB	0.16 (0.11 - 0.22) bB	0.51 (0.33 - 0.69) bA

Lowercase letters compare cementing materials for the same toothbrushing cycle. Upper case letters compare toothbrushing cycles for the same cementing material.

### Roughness Profile

Table 2 presents the roughness profile results for the cementing materials at baseline, 20.000, and 60.000 toothbrushing cycles. As for the surface roughness, the roughness profile also increased with the increase in the number of toothbrushing cycles. Regarding the resin cement-ceramic interface, Z100 presented a lower roughness profile than that obtained for REU2, while REXU did not differ from Z100 and REU2 at the baseline. For 20,000 cycles, REU2 showed the highest roughness profile, while for 60,000 cycles, no statistical difference was found among cementing materials. Regarding the resin cement-enamel interface, REU2 showed the highest roughness profile at the baseline and both toothbrushing cycles, while Z100 and REXU did not differ between them.

**Table 2:** Roughness profile means (Rv, in µm) for the cementing materials (95% CI).

	Resin Cement-Ceramic			Resin Cement-Enamel		
Materials	Baseline	20,000 cycles	60,000 cycles	Baseline	20,000 cycles	60,000 cycles
REXU	0.04 (0.03 - 0.05) abB	0.10 (0.07 - 0.13) bA	0.18 (0.12 - 0.24) aA	0.03 (0.02 - 0.05) bB	0.09 (0.06 - 0.13) bA	0.13 (0.09 - 0.17) bA
REU2	0.07 (0.05 - 0.09) aB	0.18 (0.12 - 0.24) aA	0.28 (0.19 - 0.37) aA	0.11 (0.07 - 0.15) aB*	0.18 (0.12 - 0.24) aAB	0.26 (0.18 - 0.35) aA
Z100	0.04 (0.02 - 0.05) bC	0.07 (0.05 - 0.09) bB	0.17 (0.12 - 0.23) aA	0.04 (0.02 - 0.05) bB	0.07 (0.05 - 0.09) bA	0.13 (0.09 - 0.17) bA

Lowercase letters compare cementing materials for the same toothbrushing cycle and interface. Upper case letters compare toothbrushing cycles for the same cementing material and interface. (*) Differ from "Resin Cement-Ceramic interface" for the same cementing material and toothbrushing cycle.

### Volume Loss and Confocal Microscopy Images

The volume loss of cementing materials after toothbrushing is present in Table 3. Regarding the resin cement-ceramic interface, Z100 showed the lowest volume loss at 20,000 toothbrushing cycles, while the REXU was the lowest and REU2 was the highest at 60,000 cycles. For the resin cement-enamel interface, Z100 also showed the lowest volume loss at 20,000 toothbrushing cycles and RXU and REU2 did not differ between them. For 60,000 cycles, REXU and Z100 presented lower volume loss than that obtained for REU2. The increase in the number of toothbrushing cycles increased the volume loss only for the REU2 and Z100 cementing materials.

**Table 3:** Volume loss (x 10^5^) means (in µm^3^) for the cementing materials (95% CI).

	Resin Cement-Ceramic (CC)			Resin Cement-Enamel (CE)	
Material	20,000 cycles	60,000 cycles	20,000 cycles	60,000 cycles
REXU	2.6 (1.5 - 3.7) aA	2.4 (1.4 - 3.4) cA	2.2 (1.3 - 3.1) aA	2.1 (1.2 - 3.0) bA
REU2	3.1 (1.8 - 4.4) aB	15.9 (9.2 - 20.3) aA	1.9 (1.1 - 2.6) aB	14.3 (8.3 - 20.3) aA
Z100	0.4 (0.2 - 0.6) bB	5.9 (3.4 - 8.4) bA	0.4 (0.3 - 0.6) bB	3.6 (2.1 - 5.2) bA

Lowercase letters compare cementing materials for the same toothbrushing cycle and interface (CC or CE). Upper case letters compare toothbrushing cycles for the same cementing material and interface (CC or CE). There is no difference between CT and CC interfaces.

**Figure 1:**
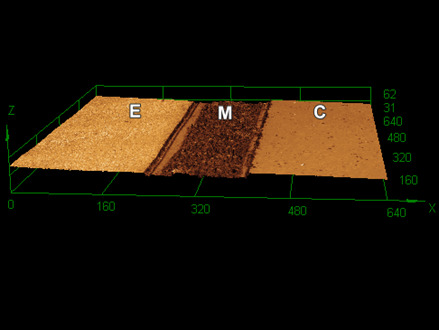
Three-dimensional confocal image showing the enamel (E), the REXU cementing material (M), and the ceramic (C) after 60.000 toothbrushing cycles.

**Figure 2:**
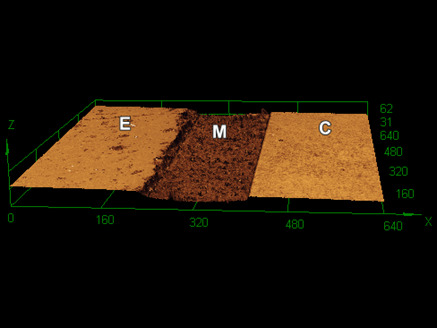
Three-dimensional confocal image showing the enamel (E), the REU2 cementing material (M), and the ceramic (C) after 60.000 toothbrushing cycles.

**Figure 3:**
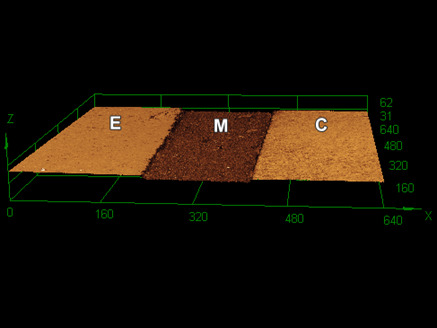
Three-dimensional confocal image showing the enamel (E), the Z100 cementing material (M), and the ceramic (C) after 60.000 toothbrushing cycles.

Figures 1 to 3 show 3D images of the topography of the samples after 60,000 toothbrushing cycles. REXU (Figure 1) and Z100 (Figure 3) resin-based cement seemed to lose less material volume than REU2 (Figure 2). Toothbrushing increased the surface roughness causing filler particles exposal due to the wear of the superficial layer of the cementing materials. The thickness of the cementing materials between the ceramic and the dental enamel was approximately 180 µm.

## Discussion

The first research hypothesis stating that the simulated toothbrushing does not affect the surface roughness of cementing materials was rejected because the abrasion effects caused by toothbrushing until 60.000 cycles significantly increase the surface roughness for all materials. The 20,000 and 60,000 toothbrushing cycles used in this study correspond to approximately two and six years of toothbrushing, respectively ^16^,^17^, which may help in understanding the longevity of feldspathic ceramic restorations bonded to tooth enamel ^18^.

The heating of Z100 restorative composite reduces its viscosity, allowing it to serve as a cementing material ^11^. Z100 composite was chosen in this study because it is a cheap commercial dental composite and it has been commonly used by clinicians as a luting material and tested by researchers showing proper results ^19^,^20^. The surface roughness, roughness profile, and volume loss results for this restorative composite were better or similar to other cementing materials tested (REUX and REU2).

Current restorative composites present lower polymerization shrinkage compared to the composites of the 80s and 90s. Also, they reach an adequate degree of conversion and mechanical strength due to the higher amount of filler particles and the presence of high molecular weight and new monomers in their compositions ^21^. Thus, the restorative resin composite layer at the cementation area might be more resistant to intraoral challenges ^12^.

Besides reducing the viscosity, pre-heating of conventional restorative composites and bulk-fill resins before light-activation decreases polymerization-induced shrinkage forces without compromising the degree of conversion ^10^ which might improve the marginal adaptation of the indirect restorations ^11^. A previous study tested the film thickness of various heated restorative resin composites (69ºC) and the results did not identify the influence of filler loading and viscosity. Preheating can reduce the composite's viscosity between 47% and 92%, which is generally more viscous than the flowable ones ^14^. As a clinical limitation, preheated resin composites have a short working time, because the composite does not keep the heating and changes quickly to a high-viscosity state, when it is no longer recommended to be used as cementing material ^13^.

The REXU resin cement was used in combination with Scotchbond Universal adhesive, similar to Z100. The manufacturer recommends using REXU resin cement specifically with the Scotchbond Universal adhesive and this cementing system can be used in two activation modes: chemical and light-activation (or dual-cure). The chemical mode tries to ensure sufficient polymerization of resin cement in regions where there is no light exposure. However, studies have shown that the light-activation of resin cement can increase its degree of conversion and wear resistance. Thus, the light-activation gives the best curing conditions for cementation materials ^22^ ^), (^ ^23^ ^)(^ ^24^.

REUX and heated Z100 restorative composite presented similar results in this study because these resin-based materials present similar resin matrix (methacrylate monomers) and filler content (silanized glass filler particles). Their surface roughness and roughness profile did not differ between them, regardless of the number of toothbrushing cycles. For volume loss at 60,000 cycles, REUX and Z100 presented lower volume loss than that obtained for REU2. REXU did not differ from REU2 when analyzed at 20,000 cycles and it was the only cementing material that did not show a significant increase in volume loss with an increase in the number of toothbrushing cycles.

The second hypothesis that the roughness profile varies among cementing materials after toothbrushing was accepted because significant differences were obtained among cementing materials at baseline, 20.000, and 60.000 cycles. In general, REXU and Z100 showed a lower roughness profile than REU2 after toothbrushing cycles, because of the exposure of filler particles of REU2 following the toothbrushing, which are larger ^8^ than those of REXU and Z100 resin cements. The third hypothesis that the toothbrushing cycles induce the volume loss of the cementing materials was accepted only for REU2 and Z100 because they showed significantly higher volume loss at 60,000 cycles compared with 20,000 cycles.

 REU2 material presented highest the mean of surface roughness at baseline (0.32 µm) and after 20,000 toothbrushing cycles (0.49 µm). In general, the highest roughness profile was observed for this self-adhesive resin cement. For volume loss, the REU2 presented higher values than REXU and Z100 at 60,000 cycles. The higher volume loss of REU2 may be due to the lower mechanical properties presented by some self-adhesive resin cement compared to conventional resin cements ^6^.

The confocal 3D images (Figures 1 to 3) show the surface wear of cementing materials promoted by 60,000 toothbrushing cycles. The altered surface of cementing materials after the toothbrushing exposed the filler particles by the removal of the superficial layer of cementing materials. As a result, an increase in surface roughness and a change in the roughness profile were observed and reported in this study for the dentists understand the performance of different cementing materials under simulated toothbrushing. Luting material wear can lead to gap formation at the tooth-prosthesis interface and staining. Also, changes in surface roughness and topography can cause biofilm retention at the resin cement layer, since from 0.2 μm of surface roughness biofilm retention becomes more prone ^25^.

## Conclusion

The toothbrushing can alter the topography of the enamel-cementing material-ceramic bonded interface, influencing the surface roughness, roughness profile, and volume loss results of the cementing materials. Among the luting materials evaluated in this study, REXU and heated Z100 showed the best results and might present better clinical performance after approximately six years of toothbrushing.
